# Mindfulness-Based Cognitive Therapy for Cancer Patients Delivered via Internet: Qualitative Study of Patient and Therapist Barriers and Facilitators

**DOI:** 10.2196/jmir.7783

**Published:** 2017-12-18

**Authors:** Félix R Compen, Else M Bisseling, Melanie PJ Schellekens, Ellen TM Jansen, Marije L van der Lee, Anne EM Speckens

**Affiliations:** ^1^ Centre for Mindfulness Department of Psychiatry Radboud University Medical Centre Nijmegen Netherlands; ^2^ Donders Institute for Brain, Cognition and Behaviour Nijmegen Netherlands; ^3^ Helen Dowling Institute for Psycho-Oncology Bilthoven Netherlands

**Keywords:** mindfulness, psycho-oncology, cancer survivors, telemedicine, qualitative research

## Abstract

**Background:**

The number of patients living with cancer is growing, and a substantial number of patients suffer from psychological distress. Mindfulness-based interventions (MBIs) seem effective in alleviating psychological distress. Unfortunately, several cancer patients find it difficult, if not impossible, to attend a group-based course. Internet-based MBIs (eMBIs) such as Internet-based mindfulness-based cognitive therapy (eMBCT) may offer solutions. However, it is yet to be studied what facilitators and barriers cancer patients experience during eMBCT.

**Objective:**

This study aimed to explore facilitators and barriers of individual asynchronous therapist-assisted eMBCT as experienced by both patients and therapists.

**Methods:**

Patients with heterogeneous cancer diagnoses suffering from psychological distress were offered eMBCT. This 9-week intervention mirrored the group-based MBCT protocol and included weekly asynchronous written therapist feedback. Patients were granted access to a website that contained the eMBCT protocol and a secured inbox, and they were asked to practice and fill out diaries on which the therapist provided feedback. In total, 31 patients participated in an individual posttreatment interview on experienced facilitators and barriers during eMBCT. Moreover, eight therapists were interviewed. The data were analyzed with qualitative content analysis to identify barriers and facilitators in eMBCT.

**Results:**

Both patients and therapists mentioned four overarching themes as facilitators and barriers: treatment setting (the individual and Internet-based nature of the treatment), treatment format (how the treatment and its guidance were organized and delivered), role of the therapist, and individual patient characteristics.

**Conclusions:**

The eMBCT provided flexibility in when, where, and how patients and therapists engage in MBCT. Future studies should assess how different eMBCT designs could further improve barriers that were found.

## Introduction

Cancer poses a major psychological challenge for individuals. A meta-analysis of psychiatric disorder in oncological and hematological settings yielded a prevalence of psychiatric disorder of 30% to 40% [1]. In the coming decades, a great increase is expected in the number of people living with cancer [2]. This means that a growing number of cancer patients are in need of effective and accessible psychological treatment.

Mindfulness-based interventions (MBIs) such as mindfulness-based stress reduction (MBSR) [3] and mindfulness-based cognitive therapy (MBCT) [4], the latter more oriented toward those in need of clinical psychological treatment, could be viable intervention options for cancer patients. Mindfulness is defined as follows: “paying attention, on purpose, in the present moment and nonjudgmentally” [5]. Its practice enables participants to recognize habitual, conditioned modes of reacting, and to make a radical shift in how they relate to their thoughts, feelings, and body sensations, as well as to outer circumstances [6], such as when coping with cancer.

Evidence for the effectiveness of MBIs for cancer patients has rapidly expanded. In 2015, an overview including six systematic reviews in heterogeneous cancer patients demonstrated significant small to moderate effects on various psychosocial outcomes in cancer patients [7]. In addition, studies confirmed these effects at longer-term follow-up [8,9]. Moreover, studies demonstrated that the effect of MBIs in breast cancer patients might be mediated by nonreactivity, reduced catastrophizing, and increased self-kindness [10,11]. Notwithstanding the potential of MBIs, several cancer patients encounter practical and psychosocial barriers that hamper access and participation in psychological treatments such as MBIs. These barriers include cancer-related illness, fatigue, limited mobility or disability, limited transportation options, and time constraints [12,13]. Internet-based interventions, such as Internet-based MBIs (eMBIs), may offer solutions to these problems. Mobile MBI apps have already demonstrated their potential [14]. Internet-based interventions are easily accessible, available 24×7, save traveling time, and could be less costly [15]. Evidence of over 100 well-controlled trials suggests that Internet-based treatments can be as effective as group-based psychological treatments for a wide range of psychiatric and somatic conditions [16].

Moreover, a previous review suggests that eMBIs may be helpful in alleviating symptom burden of patients with physical health conditions, particularly when the eMBI is tailored to specific symptoms [17]. A total of 16 studies examining eMBIs for people with chronic physical health conditions were reviewed, of which two specifically targeted cancer patients. A randomized controlled trial (RCT; n=62) investigated the quantitative feasibility of Internet-based MBSR for cancer patients (mindfulness-based cancer recovery [MBCR]) [13]. The Internet-based MBCR (eMBCR) consisted of synchronous videoconferencing sessions. Feasibility targets for recruitment and adherence (5% response rate, 30% eligible, and 85% consented) were achieved, and patients considerably improved on mood disturbance, stress symptoms, spirituality, and acting with awareness in the Web-based group relative to waitlist controls. Results suggested that eMBCR led to improved energy while also inducing relaxation [18]. In addition, an uncontrolled cohort study (n=257) of severely fatigued cancer patients evaluated an Internet-based mindfulness-based cognitive therapy (eMBCT) [12]. In total, 34.6% (89/257) of the patients showed clinically relevant improvement in fatigue severity and 61.8% (159/257) of the patients adhered to treatment. In sum, evidence for eMBIs in cancer is scarce, but the first results seem promising.

However, how to optimally deliver eMBIs remains unknown [17]. It is unclear whether either synchronous (real time, eg, instant messaging or videoconferencing) or asynchronous (delayed, eg, email or message boards) is to be preferred. Patients are supposed to engage in an experiential inquiry-based learning process together with the therapist in eMBIs [6], but it is unknown whether such an experiential inquiry-based learning process is at all possible in an asynchronous format. In addition, it is unclear whether either facilitated (guided) or self-directed eMBIs are to be preferred. It is argued that the therapists’ capacity to embody qualities and attitudes of mindfulness in the process of teaching is vital for effective delivery of MBIs [6]. Guidance seems to be a beneficial feature of Internet-based interventions in general [19], and exploratory subgroup analyses of a systematic review indicated higher effect sizes of stress and mindfulness skills for guided than unguided eMBIs [15]. However, a previous review also provided some initial support for unguided eMBIs [20]. In short, the question of which eMBI delivery format is preferable in terms of program adherence, mindfulness skills, and outcome improvement needs further investigation [17].

Previously, a qualitative study has provided important perspectives for examining the user experience in an MBI. In a qualitative study of an eMBI for recurrent depression, patients identified aspects such as flexibility and reduced cost, as well as the need for support in time management [21]. Qualitative information on how patients and their therapists experience eMBCT could identify barriers and facilitators, and inform us whether it is possible to design useful, user-friendly, and effective eMBIs for cancer patients and where to improve delivery mode and design if necessary and possible. Therefore, the aim of this study was to gain understanding of the experienced facilitators and barriers of asynchronically delivered eMBCT in a sample of heterogeneous cancer patients and their therapists.

## Methods

### Study Population and Procedure

The patients of this study took part in a 3-armed trial on the (cost-) effectiveness of MBCT for distressed cancer patients (Clinicaltrials.gov no. NCT02138513) [22]. Patients were randomized to either eMBCT, group-based MBCT, or treatment as usual. The RCT is described in more detail in a protocol paper [22]. Patients for this trial were mainly recruited via online media (26.9%, 66/245), patient associations (17.6%, 43/245), and participating mental health care centers (16.7%, 41/245). In total, 245 cancer patients with any tumor type and any stage of disease scoring 11 or higher on the Hospital Anxiety and Depression Scale were randomized. The local ethics committee approved this study (CMO Arnhem Nijmegen 2013/542).

### Qualitative Assessments: Semistructured Interviews (Patients) and Focus Group (Therapists)

Both patients randomized to eMBCT and their therapists were invited by the researcher to talk about the following questions:

- How did you experience the eMBCT?
- What facilitated and what impeded your participation in eMBCT?
- How did you experience the relationship with the therapist or patient?
- How would you improve the eMBCT?

The abovementioned questions were followed by specific probes *.* Questions were asked in an open nondirective manner, allowing participants to speak freely about their experiences.

Patients were interviewed via telephone or in person within 3 months after eMBCT treatment completion or dropout. Patients were purposefully sampled to gather an even distribution of completers versus noncompleters and breast cancer versus other tumor types. Patient interviews were conducted by FC and EJ. FC is a PhD student with an MSc degree in behavioral science with no prior experience in qualitative research. He was the trial coordinator for the larger RCT [22], and had conducted baseline and posttreatment research interviews conducting the posttreatment interviews. EJ is a psychologist and mindfulness teacher with an MSc degree in psychology with prior experience with qualitative research.

Therapists were invited for a focus group interview during the last plenary therapist supervision session approximately 3 months after completing the last MBCT. Both the patient interviews and therapist focus group started by explaining confidentiality and the explorative nature of the interview. AS and ML conducted the focus group interviews. AS is a professor of psychiatry in the role of principle investigator of the larger RCT [22], with experience in several qualitative research projects in MBIs. ML is a senior researcher and clinical psychologist in the role of principle investigator of the larger RCT [22], with experience in several qualitative research projects on MBIs.

### Data Analysis

We used conventional qualitative content analysis to analyze the data in which coding categories are derived directly from the text data [23]. We used ATLAS.ti version 7.1 software (Scientific Software Development GmbH, Berlin, Germany). Analysis started as soon as the first interview was conducted and continued with each additional interview. Interviews were transcribed verbatim, and each transcript was coded by 2 independent researchers (FC and EJ) to identify facilitators and barriers. Coding was performed as closely related to the patient’s words as possible to minimize subjectivity. After 5 interviews, FC and EJ compared codes with each other, and a common coding scheme was developed. Remaining transcripts were coded using this common coding scheme and earlier transcripts were recoded. New codes were added when data were encountered that did not fit in the existing coding scheme. After 12 interviews, a larger group of researchers (FC, EJ, MS, ML, and AS) discussed all data within the coding scheme. Some codes were combined during this process, whereas others were split in subcategories. After 31 interviews, no new codes were added and it was concluded that saturation had been reached. All codes referring to the same phenomenon were grouped in a hierarchical structure in subcategories, and subcategories in themes by FC and EJ. The group of researchers (FC, EJ, MS, ML, and AS) subsequently discussed this classification until reaching consensus.

### Intervention and Therapists

The eMBCT was based on the MBCT protocol for recurrent depression published by Segal et al [4]. The content was adapted to cancer patients by particularly tailoring the psychoeducation (eg, managing cancer-related fatigue, pain, fear of cancer recurrence, and effects of cancer on partner communication) and movement exercises to their needs [24].

**Figure 1:**
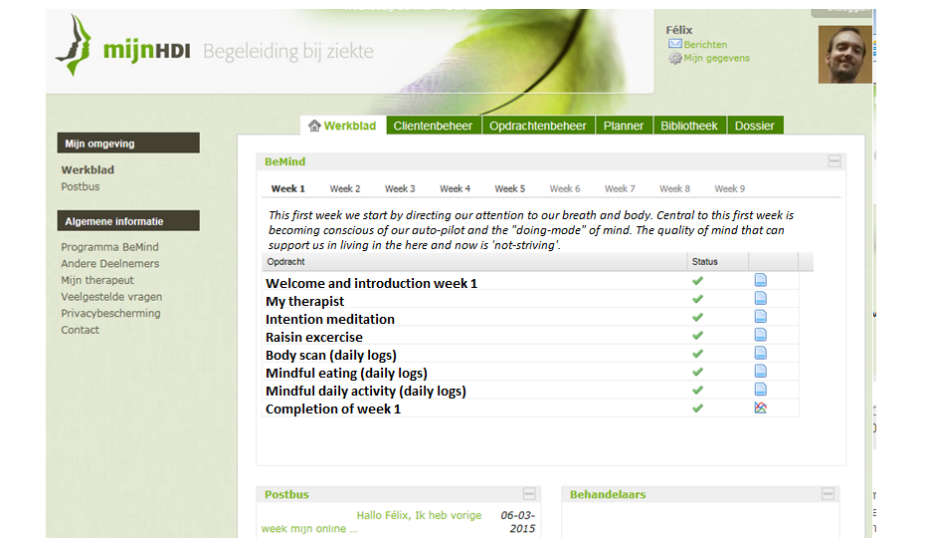
The eMBCT dashboard containing the programme overview.

**Figure 2:**
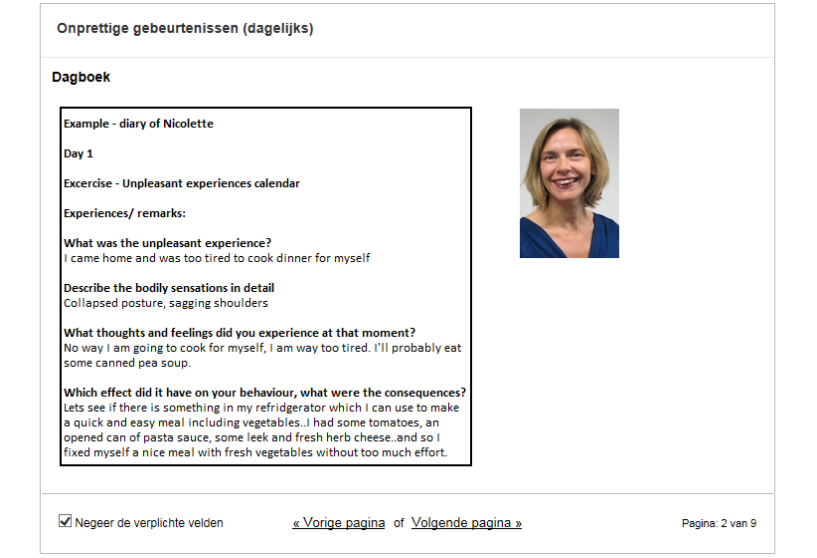
A fellow (fictional) participants' diary entry.

**Figure 3:**
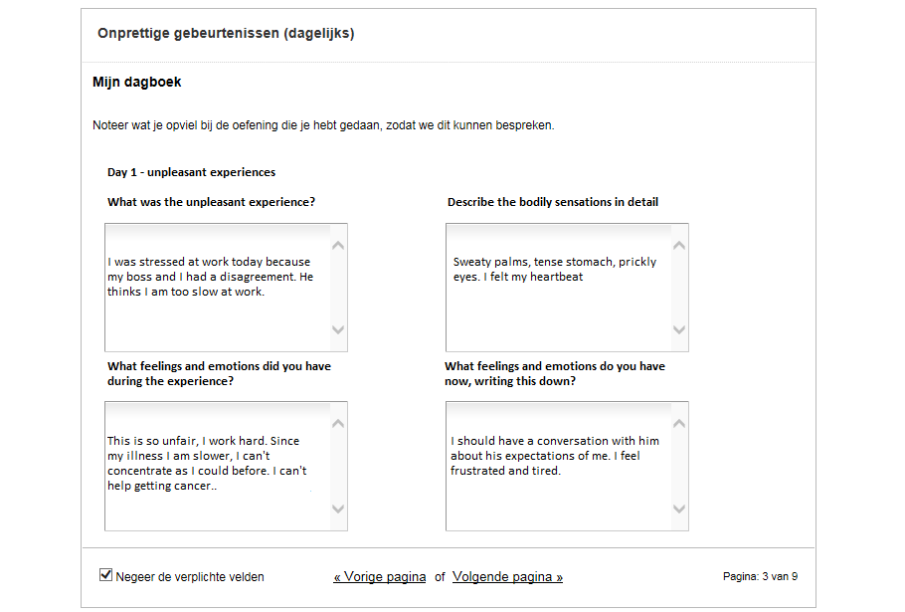
Example of an online eMBCT diary form accompanying one of the homework assignments.

**Figure 4:**
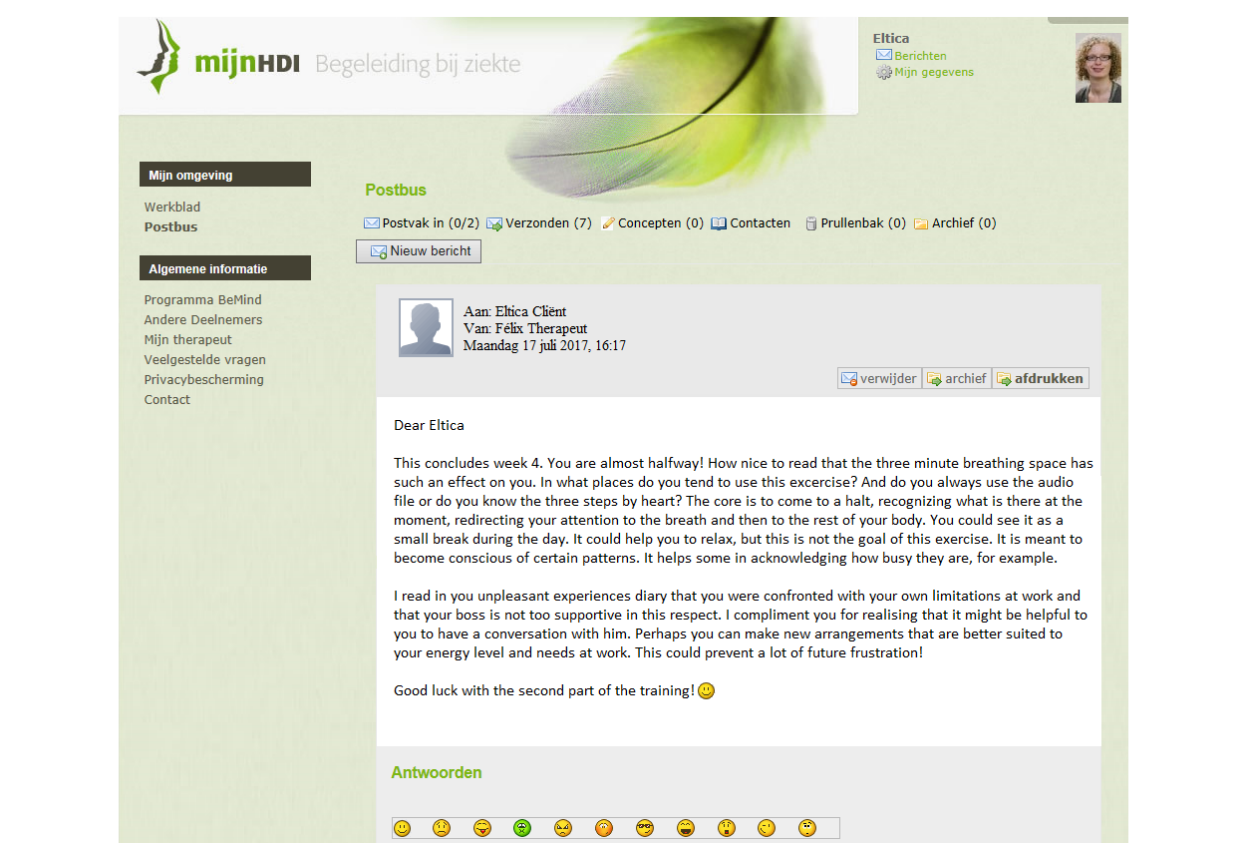
Written therapist feedback via the secured messaging inbox.

The eMBCT was mainly text-based and included asynchronous interaction with a therapist similar to the study of Bruggeman-Everts et al [12]. Patients were granted access to a website divided into a workspace containing 9 sessions (8 weeks + 1 full-day silent retreat) and a secured inbox. The therapist initiated the eMBCT by sending a welcome message to the patient. When patients logged-in, they were presented with the overview of all information and assignments due for that week (Figure 1). Each session contained an introductory text, and daily formal (eg, sitting meditation) and informal exercises (eg, awareness of everyday activities) with guided audiotaped files and accompanying diaries. Sessions also contained other home practice such as the pleasant or unpleasant events’ diary. The eMBCT was performed individually. To demonstrate the rationale and possible obstacles of each exercise, patients were provided with experiences of other (fictional) patients (Figure 2). Patients were asked to practice and complete the diaries on a daily basis (Figure 3). The therapist provided written feedback on their progress via the secured inbox on a prearranged day of the week (Figure 4). Next week’s session only became available after completing the previous session. Patients always had access to their therapist via the inbox. Patients and therapists were notified via their regular email when they received a message via the secured inbox.

We defined adherence as having attended ≥4 sessions. Therapists without prior eMBCT experience were provided with guidelines and were supervised by more experienced eMBCT therapists. See Table 1 for therapists’ characteristics. All therapists fulfilled the advanced criteria of the Association of Mindfulness Based Teachers in the Netherlands and Flanders, which are in concordance with the UK Mindfulness-Based Teacher Trainer Network Good Practice Guidelines for teaching mindfulness-based courses [25].

**Table 1:** Demographical characteristics of Internet-based mindfulness-based cognitive therapy therapists.

Variable (N=8)	Mean (SD)	n (%)
Age, years	55.50 (7.2)	
Gender, female		6 (75)
Years of experience in teaching MBCT^a^	8.75 (2.7)	
Prior experience with eMBCT^b^		4 (50)

^a^MBCT: mindfulness-based cognitive therapy.

^c^eMBCT: Internet-based mindfulness-based cognitive therapy.

## Results

### Sample

Out of the 125 patients randomized to eMBCT, 45 were invited for a posttreatment interview. In total, 12 patients declined and 2 recordings failed. As a result, 31 interviews were used in the qualitative analysis. Interviews lasted from 5 to 25 min. Out of the patients interviewed, 14 had participated in 4 or more sessions of eMBCT, 10 had attended less than 4 sessions of eMBCT, and 7 had not started at all. See Table 2 for patient characteristics.

A total of 11 out of 12 eMBCT therapists were invited for a focus group interview after completion of all eMBCTs. Out of these 12 therapists, 7 therapists agreed to participate and 1 therapist agreed to provide an individual interview with FC for scheduling reasons. Therapists declined either because of having provided too few individual online treatments to share experiences (n=1) or because of scheduling reasons (n=2). The focus group interview lasted for 90 min. The single individual therapist interview lasted for 25 min. The final sample of therapists included both therapists who had experience with online mindfulness before this project (n=4) and therapists who had no prior experience with online mindfulness before this project (n=4). See Table 2 for therapist characteristics.

All patient facilitators (Textbox 1), patient barriers (Textbox 2), therapist facilitators (Textbox 3), and therapist barriers (Textbox 4) could be divided into the following four themes: treatment setting, treatment format, role of the therapist, and patient characteristics. First, patients’ facilitators and barriers are presented per each theme. Then, therapists’ facilitators and barriers are presented per each theme.

### Patients

#### Patient Theme 1: Treatment Setting—Facilitators

Treatment setting concerned subthemes on the external conditions of the eMBCT: flexibility of timing, the individual nature, and the home practice environment of the training.

##### Time Management

It was considered convenient to be able to manage your own time schedule, which increased treatment receptivity. One patient stated the following:

Because you can start when you are ready and have the peace of mind for it, you can absorb it much better, because you actually want to at that moment.

Female breast cancer patient (curative), 65 years, completer

##### Individual Setting

A patient indicated that the individual setting facilitated a sense of autonomy that helped in taking care of himself:

I didn’t feel like doing the movement exercises. In a group setting I would have had to explain myself, so you are more inclined to go along with the group. But now, being on my own, I carried full responsibility for my own actions. Getting this space felt comfortable, because there were moments at which the therapy really asked a lot of me. At those times I could allow myself to take a time out and decide when I wanted to continue again.

Male prostate cancer patient (palliative), 65 years, completer

Furthermore, it was considered to be facilitating not to be confronted with other patients’ cancer stories. One patient stated the following:

This only was about me and I didn’t have to spend energy on someone else’s story.

Female breast cancer patient (curative), 27 years, completer

##### Home Setting

Being able to complete the sessions and exercises in your own home environment and not having to travel was appreciated. One patient stated:

For me, it was ideal because I knew that the group-based MBCT would take place at [the mental health institute] and it was impossible to reach by public transport.

Female breast cancer patient (curative), 27 years, completer

**Table 2:** Demographic and clinical characteristics of Internet-based mindfulness-based cognitive therapy patients.

Variable (N=31)			Mean (SD)	n (%)
Age, years			53.0 (12.3)	
Gender, male				6 (19)
**Education level**				
	Secondary			14 (45)
	Vocational or university			17 (55)
Time since diagnosis			3.2 (2.7)	
**Cancer diagnosis**				
	Breast			16 (52)
	Other			15 (48)
**Cancer treatment intent**				
	Curative			24 (77)
**Active cancer treatment**				
	Yes			11 (35)
Psychological distress, HADS^a^			16.2 (7.1)	
**MBCT**^b^ **adherence**				
	Completer			14 (45)
	**Dropouts**			10 (32)
		Other priorities		4 (40)
		Too difficult		3 (30)
		Too intensive		1 (10)
		Illness		1 (10)
		Missed peers		1 (10)
	**No start**			7 (23)
		Wanted MBCT		3 (43)
		Illness		1 (14)
		Other priorities		1 (14)
		Could not log in		1 (14)
		Needed mental health services		1 (14)

^a^HADS: Hospital Anxiety and Depression Scale.

^b^MBCT: mindfulness-based cognitive therapy.

**Patient facilitators across four themes and subthemes.**

Theme 1: treatment setting

- Time management
  - Program at own time improves receptivity
- Individual setting
  - Sense of autonomy
  - Not having to cope with other patients’ stories
- Home setting
  - Not having to travel

Theme 2: treatment format

- Website
  - Clear and easy to navigate
  - Privacy precautions
- Diaries
  - Rereading own notes
  - Stimulated reflection

Theme 3: role of the therapist

- Practical guidance
  - Clarifying practical matters
- Mindfulness
  - Deepened understanding
  - Embodiment stimulated practice

Theme 4: patient characteristics

- Writing fluency
  - Written expression in describing experiences
- Curiosity
  - Curiosity stimulated perseverance

**Patient barriers across four themes and subthemes.**

Theme 1: treatment setting

- Time management
  - Responsibility for time management
- Individual setting
  - No learning from peer group
- Home setting
  - Lack of privacy in own home
- Illness barriers
  - Cancer-related reading impairments
- Lack of information
  - Lack of information before start

Theme 2: treatment format

- Website
  - Complicated
- Diaries
  - Complicated to fill out
  - Obligatory nature was burdensome
  - Describing experiences was confrontational

Theme 3: role of the therapist

- Asynchronicity
  - No dialogue emerging
- Frequency
  - Wished more frequent feedback

Theme 4: patient characteristics

- Writing fluency
  - Lack of verbal fluency made diaries difficult

#### Patient Theme 1: Treatment Setting—Barriers

##### Time Management

Responsibility for your own time management was mentioned as a barrier because it required a lot of self-discipline. One patient stated:

What I like about it is that I can manage my own time which went very well the first couple of weeks. After a while some chores interrupted me and then at the end of the day I realized: I still have to practice. Sometimes I did not do it anymore and sometimes I did. So you have to be very disciplined to stick to the schedule.

Female cervical cancer patient (curative), 46 years, completer

##### Individual Setting

Patients described the lack of a group setting as a drawback of the intervention. They missed the peer support and the ability to learn together in the eMBCT. One patient stated:

I am a rational being. In a group there are always others who help me to unravel my emotions. This helps me. And I know, when I sit behind my computer my autopilot turns on and the treatment becomes a rational, experimental exercise.

Female breast cancer patient (curative), 54 years, no start

##### Home Setting

Other patients mentioned that they felt less comfortable having to do exercises at home, not having the privacy they needed. One patient stated:

I practiced in my home office, but that room is connected to my living room. I found it uncomfortable to practice with my husband around, and even though he would never be eavesdropping, I felt restricted in doing certain exercises.

Female colon cancer patient (curative), 60 years, completer

##### Illness Barriers

One patient indicated that her cancer type caused her to have trouble reading. As the eMBCT was mainly text-based, this was a problem to her. She stated the following:

It was mainly physical, I didn’t have the energy and my vision is in such a bad state. Even with medication, my vision is bad. And my eyes itch and burn and hurt.

Female bone marrow cancer patient (palliative), 55 years, no start

##### Lack of Information

Moreover, patients indicated that they would have wished more information on the way the platform and course were organized before the start of the training. One patient stated:

Expectation management would have helped a lot, I had a very brief instruction. And I have to choose where to put my energy into. What is expected of me, can I handle it, does it fit in my planning?

Female breast cancer patient (curative), 54 years, no start

#### Patient Theme 2: Treatment Format—Facilitators

The treatment format theme included codes on the facilitators and barriers of the means by which the eMBCT was internally organized and delivered.

##### Website

The website was accessible and navigating throughout the website was easy. One patient stated:

Opening the exercises and the way [the website] guided you through the structure was easy.

Male skin cancer patient (curative), 61 years, completer

Moreover, patients valued the privacy precautions and indicated that the website felt safe. One patient stated:

I thought it was neat that I could see who visited my profile. In my case it was only my therapist according to the system, so I presume that the system is right, but it felt well taken care of.

Female melanoma patient (curative), 33 years, completer

##### Diaries

The diaries proved to be of value for patients because it enabled them to read back and learn from their own experiences. One patient stated:

In my own [diaries] I looked back to see what my experiences were yesterday, or how did I handle this last time?

Female melanoma patient (curative), 33 years, completer

Patients also indicated that having to write stimulated reflection upon experiences. One patient stated:

Writing about my feelings was different from when I would have talked about it. It was more reflective, less spontaneous. I noticed that when I mailed I checked it again and again and added a few things. This really was an advantage. It really made me think about what I felt and experienced. Because of the writing itself this really hit me.

Female breast cancer patient (curative), 61 years, dropout

#### Patient Theme 2: Treatment Format—Barriers

##### Website

The website was complicated to some patients. One patient stated:

The website and its explanation was not really user friendly. There were many steps you had to take before you could do what you actually had to do.

Female breast cancer patient (palliative), 49 years, dropout

##### Diaries

A patient mentioned that the diaries were complicated to fill out:

I got the message that some fields still needed to be filled out. In general, I couldn’t find where to fill out what in the diaries and it made me quit.

Male prostate cancer patient (palliative), 78 years, dropout

Patients thought it was burdensome that the diaries were obligatory. One patient stated:

It was so much. Filling out the diaries every day [...]. I subscribed for a mindfulness course because I didn’t feel well and all of a sudden, you have this huge obligation.

Male palate cancer patient (curative), 30 years, dropout

The diaries were also considered quite confronting at times. One patient stated:

When you had a negative experience, filling out the diary made me revive the negative moment.

Female cervical cancer patient (curative), 50 years, dropout

#### Patient Theme 3: Role of the Therapist—Facilitators

This theme included codes on the role of the therapist and the way the therapist facilitated or hindered participating in eMBCT.

##### Practical Guidance

Patients indicated that the therapist was often able to clarify practical aspects that were unclear. One patient stated:

I always want to do things right, and I wasn’t sure about how I did the meditation exercises in the beginning. Is this the way I am supposed to do this? So after a while I just mailed my therapist asking questions about the how and what of exercises, and I got a prompt reply most of the times.

Female melanoma patient (curative), 33 years, completer

##### Mindfulness

The ways in which therapists provided feedback enriched patients’ understanding of underlying mindfulness values, such as the mild and nonjudgmental attitude. One patient stated:

(My therapist) was very patient and gave me all the space I needed [...]. She was like this all the time, in everything she did, not forcing, but stimulating me. “Do it for yourself when you do the exercises. If you do them, you could benefit a lot.” This made me feel more connected.

Female breast cancer patient (curative), 52 years, completer

The embodiment of mindfulness values, such as the nonjudgmental attitude, supported and motivated patients to practice with the right intentions. One patient stated:

My therapist struck me as very mild. “Don’t force yourself, be gentle,” that certainly stood out. I don’t know how she would have been if I hadn’t practiced as much, but she was gentle with me.

Female melanoma patient (curative), 33 years, completer

#### Patient Theme 3: Role of the Therapist—Barriers

##### Asynchronicity

The asynchronous nature of the feedback proved to be a barrier. According to the patient, the written feedback of therapist did not seem to encourage a dialogue but rather seemed limited to giving responses to questions. One patient stated:

Suppose I tell you I found the exercise uncomfortable. I then send you a message saying “I found it uncomfortable.” Only after 3 days I then get a reply “What was uncomfortable? Can you specify what you mean?” I then specify what I mean in another message. You keep sending messages back and forth over a period of time. If you have a conversation with someone, you have direct interaction. It is a totally different mode of communication. In a business context I think messaging is fine. In this context it was unhelpful.

Female breast cancer patient (curative), 56 years, dropout

##### Frequency

As the therapist provided feedback on past weeks’ diaries, sometimes questions were left unanswered for a whole week. Some patients would have liked to have more frequent feedback. One patient stated:

Just two three times a week a brief moment of contact saying “how are you”?

Female cervical cancer patient (curative), 50 years, dropout

#### Patient Theme 4: Patient Characteristics—Facilitators

Individual characteristics seemed to influence the fit between patient and eMBCT.

##### Writing Fluency

The ability to express themselves in writing was very helpful for some to give words to their subjective experiences and to ask for clarification to the therapist if it was necessary. One patient stated:

I am an easy writer, which perhaps set my experience apart from others. I can imagine that if you have a hard time expressing what you do and feel it would be different.

Female breast cancer patient (curative), 61 years, dropout

##### Curiosity

Curiosity sparked some to look beyond initial difficulties and to persevere in times of lack of motivation. One patient stated:

I think I was curious about the coming exercises. Maybe those will be more pleasant to do. This made me continue for a few more weeks.

Female colon cancer patient (curative), 60 years, completer

#### Patient Theme 4: Patient Characteristics—Barriers

##### Writing Fluency

The heavy reliance on writing skills was a barrier to some patients. One patient stated:

I liked doing the exercises, but having to write down my experiences on a daily basis [...], to sit down and write it all down, it put me off. For whom am I doing this?

Female breast cancer patient (curative), 51 years, completer

### Therapists

Facilitators and barriers experienced by therapists are depicted in Textboxes 3 and 4.

**Therapist facilitators across four themes and their subthemes.**

Theme 1: treatment setting

- Timing
  - Flexibility
- Individual setting
  - Tailoring to patient
  - Better suited to some patients

Theme 2: treatment format

- Asynchronicity
  - More time for reflection
- Schedule
  - Maintaining a schedule prevents dropout
- Writing
  - Stimulated reflection
  - Becomes more goal oriented
- Anonymity
  - Stimulates openness

Theme 3: role of the therapist

- Feedback
  - Providing group context
  - Provides reassurance
  - Personalizing training

Theme 4: patient characteristics

- Self-discipline
  - Supporting self-sufficiency

**Therapist barriers across four themes and their subthemes.**

Theme 1: treatment setting

- Timing
  - Larger time investment
  - More flexibility warranted
- Individual setting
  - No modeling by peers
  - Elaboration on personal themes

Theme 2: treatment format

- Asynchronicity
  - No present moment experiences
  - Difficulty to maintain continuity
- Technical issues
  - Technical issues cause delay
- Writing
  - No nonverbal communication
  - Limited in therapeutical repertoire
  - Lack of understanding not readily apparent

Theme 3: role of the therapist

- Feedback
  - Empty diaries impair feedback
  - More explicit checking and self-disclosure necessary
- Mindfulness
  - Embodying behind computer

Theme 4: patient characteristics

- Self-efficacy
  - Lack of self-efficacy
- Writing fluency
  - Lack of ability in written expression

#### Therapist Theme 1: Treatment Setting—Facilitators

##### Timing

Therapists welcomed the fact in that they were able to choose at what time to provide feedback, which made them adaptive to circumstances. One therapist stated:

You can provide feedback in between other chores. Sometimes you plan to give feedback from 9 to 10 and then someone enters your office. There goes your planning. I then tell myself [...] “I’ll have time at another moment.” This is an advantage, you can do it in your own time.

Female MBCT therapist, 60 years, 6 years of experience, prior eMBCT experience

##### Individual Setting

The individual nature of eMBCT allowed for tailoring to the patients’ specific circumstances and giving feedback on individual real-life examples, which increases the relevance of the feedback. One therapist stated:

In the group you only have limited amount of time during which you must touch upon the most important themes. Online I have much more choice where to provide feedback on, what it means for a specific patient to react on autopilot, and which personal themes emerge.

Male MBCT therapist, 40 years, 10 years of experience, prior eMBCT experience

Another important advantage of the individual nature of eMBCT is that it can be provided to patients who may otherwise be unsuitable for the group. Another therapist stated:

Some patients can be so disruptive in a group. They don’t get the point and only tell their own story. Sometimes you actually wished to provide someone in a group with an individual online training so you can address the individual themes.

Female MBCT therapist, 60 years, 6 years of experience, prior eMBCT experience

#### Therapist Theme 1: Treatment Setting—Barriers

##### Timing

Therapists indicated that providing feedback costs a considerable amount of time, which made it difficult for them to stick to a fixed time window. One therapist stated:

Especially in the beginning, it took me much longer. Because of asking questions, or clarifying issues. Or referring back to earlier diary entries.

Female MBCT therapist, 54 years, 12 years of experience, no prior eMBCT experience

Furthermore, therapists indicated that working online required much more flexibility and resulted in fragmentation of the times spent on eMBCT. One therapist stated:

When a patient indicates that the programme does not work, I start looking for help immediately. Even though I receive this mail outside of my regular time window for feedback.

Female MBCT therapist, 60 years, 6 years of experience, prior eMBCT experience

##### Individual Setting

Learning from fellow peer experiences in a group setting can be very helpful, and therapists felt limited in bringing in peer experiences themselves. One therapist stated:

In one-on-one contact, you can bring in experiences from other patients but to really experience them first hand provides another perspective.

Female MBCT therapist, 58 years, 11 years of experience, prior eMBCT experience

Moreover, it was often difficult to find the balance between elaboration on personal themes and the eMBCT theme. One therapist stated:

A tension emerged between someone’s personal themes and combining those with this week’s mindfulness theme. Sometimes I thought, “this patient is occupied by something entirely different.”

Female MBCT therapist, 54 years, 5 years of experience, no prior eMBCT experience

#### Therapist Theme 2: Treatment Format—Facilitators

##### Asynchronicity

Therapists and patients interacted asynchronically. This meant that according to the therapists, patients had time for reflection. One therapist stated:

Because there is some time between practice and feedback some experiences get the time to settle in. Patients can think about it, read it again, check with themselves what they experienced and how they reacted to it. This time in between could perhaps engage patients.

Female MBCT therapist, 60 years, 6 years of experience, prior eMBCT experience

Moreover, the asynchronous contact was beneficial to therapists. One therapist stated:

Sometimes my irritation causes me to cut patients off. Behind the computer I can tell myself “let’s put this to a rest for now.”

Female MBCT therapist, 54 years, 5 years of experience, no prior eMBCT experience

##### Schedule

Maintaining a fixed interaction schedule between therapist and patient was very helpful in preventing treatment dropout. One therapist stated:

When patients are able to put in work on a weekly basis and we stick to this rhythm, a kind of synchronicity emerges and assignments and my feedback to these assignments flow naturally.

Female MBCT therapist, 62 years, 10 years of experience, no prior eMBCT experience

##### Writing

Writing feedback stimulated contemplation in therapists themselves. One therapist stated:

By taking a step back I recognized, hey, it annoys me what patients write down. Or I thought by myself, “come on, start practicing.” Then I thought, “stop.” You can read back your own feedback and then think by yourself “I should not do this.”

Female MBCT therapist, 54 years, 5 years of experience, no prior eMBCT experience

Due to increasing experience, they got more efficient in their feedback over time. One therapist stated:

I became a lot more economical in my feedback over time. I tend to scan more for abnormalities or diary entries which I don’t recognize, or diary entries of which I think “this could influence dropout.” I tend to reply less, but what I say is then more relevant.

Male MBCT therapist, 40 years, 10 years of experience, prior eMBCT experience

##### Anonymity

The fact that patients were able to write about their experiences rather anonymously was helpful in opening up to experiences, which meant that in general, they shared their experiences in rather great detail. Moreover, it rendered the therapist to use patients’ own quotes. One therapist stated:

Patients think I don’t see them and they don’t see me. They tend to confide more to a diary. Sometimes they told me “I don’t know whether I should write everything down in such an uncensored manner.” And I encouraged them to do so. I sometimes used quotes from their own diaries and they asked me “Wow, did I write this down?” They sometimes used impressive words.

Female MBCT therapist, 54 years, 5 years of experience, no prior eMBCT experience

#### Therapist Theme 2: Treatment Format—Barriers

##### Asynchronicity

Therapists were unable to comment on present moment experiences. This made it difficult to communicate what mindfulness is about. One therapist stated:

The experience-driven nature, the contact when a patient says something or shows emotion with which you can work instantly, which everyone immediately feels, that is direct. And it has a lot of impact. This is why things are so slowed down in the online. You have no direct experience to work with.

Female MBCT therapist, 54 years, 11 years of experience, no prior eMBCT experience

The asynchronicity made it more difficult to maintain continuity and to prevent dropout from the eMBCT. One therapist stated:

Whenever a life event took place or I fell ill myself [...] the schedule started to get awry fairly quickly. Patients hand in their diaries too late [...] and you start hopping from miscommunication to miscommunication. In the worst case, the training gets bogged down and the output is zero.

Female MBCT therapist, 62 years, 10 years of experience, no prior eMBCT experience

##### Technical Issues

Therapists indicated that technical issues also proved to be a barrier to treatment continuity. One therapist stated:

The technical background might have been a possible reason for dropout. I thought it was difficult myself. The whole logistics of where to find what, how the site was built up, where I had to click. I didn’t think it was intuitive. It took me some time.

Female MBCT therapist, 54 years, 11 years of experience, no prior eMBCT experience

##### Writing

Therapists indicated that a major drawback of the communication in writing is the complete lack of nonverbal communication. One therapist stated:

I prefer to see someone’s nonverbal emotions. And to show that I open up. I had to think about this, how do I do this in writing? Is that even possible?

Female MBCT therapist, 58 years, 11 years of experience, prior eMBCT experience

Moreover, they sometimes felt as if their therapeutical repertoire was limited by writing. One therapist stated:

I noticed that my feedback sometimes, as it was in writing only, did not contain everything I wanted to say. My repertoire is bigger and I was not always able to use all my skills.

Female MBCT therapist, 62 years, 10 years of experience, no prior eMBCT experience

Sometimes, because of emphasis on reading and writing, it only became clear at a later stage that the patient did not fully understand everything. One therapist stated:

Sometimes patients come up with issues that have been taken care of already. Maybe because the training relies so heavily on reading and writing, patients absorb the training differently.

Female MBCT therapist, 49 years, 6 years of experience, no prior eMBCT experience

#### Therapist Theme 3: Role of the Therapist—Facilitators

##### Feedback

Therapists indicated that it was facilitating for patients that they were able to provide a group context. One therapist stated:

You can provide examples from other patients or a funny example from a group situation.

Female MBCT therapist, 60 years, 6 years of experience, prior eMBCT experience

In their feedback, they considered it motivating to provide reassurance very explicitly. One therapist stated:

In the online training I am much more complimentary for doing the exercises despite being so tired, and in the group I am much less inclined to do so.

Female MBCT therapist, 60 years, 6 years of experience, prior eMBCT experience

Therapists were also involved in making the training more personal. One therapist stated:

I make it very clear from the start that “I write this feedback to you. This is not standardized feedback,” so the patient knows he or she is dealing with an actual person. Someone actually replied “Good to know that there is a person at the other side.”

Female MBCT therapist, 54 years, 5 years of experience, no prior eMBCT experience

#### Therapist Theme 3: Role of the Therapist—Barriers

Feedback

A lack of diary entries was a turnoff for therapists in providing stimulating feedback. One therapist stated:

I noticed that it was not very stimulating when patients filled out very little. I think my own feedback will have been much shorter as well, and I much easier reverted to saying “good luck next week.”

Male MBCT therapist, 40 years, 10 years of experience, prior eMBCT experience

Therapists indicated that they experienced it as a barrier that more explicit disclosure and checking with the patient is necessary. One therapist stated:

I tell more about myself, “I recognize this when doing the body scan myself,” far more often than I used to do in a group setting, and you have to be very explicit, check and check again how things come across.

Female MBCT therapist, 58 years, 11 years of experience, prior eMBCT experience

##### Mindfulness

Therapists also stated that it was hard for them to embody mindfulness values behind the computer. One therapist stated:

When patients start to get doubtful, or skeptical about the training, the power of your presence can be really important. Not in the sense of being able to convince people but with a visible nonverbal way of saying, “everything is OK,” and showing this by being embodied. You can’t do this via the PC.

Female MBCT therapist, 62 years, 10 years of experience, no prior eMBCT experience

#### Therapist Theme 4: Patient Characteristics—Facilitators

##### Self-Discipline

Therapists indicated that for some patients, the eMBCT was partly a training in self-discipline, which supported patients’ self-sufficiency after the training. One therapist stated:

Some patients train in self-discipline. They have to, which maybe renders them more likely to continue practicing. Yes, dropout is higher, but those who do finish the training are very disciplined in doing so and did it more on their own, without the group context. More self-reliant, which is in line with mindfulness.

Female MBCT therapist, 54 years, 5 years of experience, no prior eMBCT experience

#### Therapist Theme 4: Patient Characteristics—Barriers

##### Self-Efficacy

In the eMBCT, patients need to be resolute and determined. This was mentioned as a barrier to complete eMBCT. One therapist stated:

When a patient was not able to login, the webmaster provided a link. The patient then neglected this link. If someone helps you, as a patient you must go for it and say “OK thank you, I will try again, and if it doesn’t work, I will mail you again.”

Female MBCT therapist, 62 years, 10 years of experience, no prior eMBCT experience

##### Writing Fluency

Therapists indicated that a lack of writing skills made it difficult to understand patients’ messages. One therapist stated:

Sometimes it was difficult to read past the spelling mistakes and to actually see what someone meant, and not to write down constantly “what do you mean?”

Female MBCT therapist, 58 years, 11 years of experience, prior eMBCT experience

## Discussion

### Principal Findings

The aim of this study was to gain qualitative understanding of the facilitators and barriers of eMBCT in a sample of heterogeneous cancer patients. Both eMBCT completers and dropouts participated in posttreatment interviews. Moreover, we conducted a focus group interview with eMBCT therapists. In all, this study adds to the existing quantitative evidence for eMBIs in cancer [7,8] by providing a qualitative perspective. Four overarching themes emerged, which were largely convergent between patients and therapists: treatment setting, treatment format, role of the therapist, and patient characteristics. Patients and therapists are much more flexible in when, where, and how they engage in eMBCT compared with group-based MBCT. However, most eMBCT advantages seemed to come at a price. When patients and therapists mentioned a certain aspect as facilitating (eg, the individual setting: not having to cope with other patients’ stories), they also mentioned it as barrier (no peer support).

Patients and therapists reported similar advantages and disadvantages of the timing, the individual nature, the asynchronous nature (for patients, this was detrimental to the relevance of therapist feedback, and for therapists, this was a threat to treatment continuity), the diaries, and the importance of self-discipline. The fact that so many aspects of the eMBCT were mentioned both as facilitator and barrier emphasizes the importance of offering flexibility in eMBIs [21].

There were also differences between patients and therapists. As known from a previous qualitative study on eMBCT [21], bearing the responsibility for time management was a barrier for patients. For therapists, the eMBCT seemed to require a larger time investment compared with group-based MBCT. Moreover, therapists were more concerned with the (vulnerability of) continuity of the training. They also mentioned that missing out on nonverbal information rendered them unable to spot patient withdrawal at an early stage, and to determine the reason for empty diaries. Furthermore, therapists seemed more bothered by communicating mindfulness values in eMBCT than patients. Patients specifically mentioned asynchronicity as barrier to the role of the therapist because the asynchronous communication hindered emergence of a dialogue.

### Clinical Implications

Although studies to date do not suggest that differences between how therapists handle the contact with their clients explain much variance in treatment outcome [16], the necessity of training and support for Internet-based therapists should be acknowledged. New eMBCT therapists should understand the importance of flexible availability and the dynamics of asynchronous interaction to pick up early signs of patient withdrawal.

The current eMBCT was individual, asynchronous, and therapist-assisted. One important adaptation may be to offer a peer support group [26]. The group context in MBIs supports perspective taking and the transition from personal story into investigation of common patterns of distress [6], and may foster skills relevant to valuing self and feeling close to others, which may help participants feel less isolated [21]. As a stand-alone intervention, formal online peer support group interventions for cancer patients have demonstrated preliminary feasibility and effectiveness [27].

Another consideration may be to employ a synchronous videoconferencing format [13]. This takes away the barriers associated with asynchronous delivery and may facilitate dialogue with the therapist and peer support. A possible caveat may be that videoconferencing does not alleviate the scheduling issues inherent in group eMBIs [13] and that synchronous videoconferencing solutions are technically much more demanding. An alternative to videoconferencing may be to include synchronous written conversations (or “chats”) with therapists or trained volunteers. Chats are becoming increasingly popular as Web-based mental health interventions by themselves and show inconclusive but promising evidence [28].

Eventually, one could employ a blended format, combining the advantages of Web and group-based therapy [29]. Blended eMBIs could have group-based group meetings at the start, midst, and end of the programme. The meeting at the start of the intervention could be used to address practical and technical matters, a midtreatment meeting to address common barriers experienced by patients during practice, and meeting at the end to say goodbye to each other and support patients to take responsibility for the continuation of their mindfulness practice in the future. In between group-based sessions, patients could be offered online sessions. In our view, these practical arrangements could greatly improve the acceptability and effectiveness of eMBCT.

### Research Implications

Previous studies have provided encouraging quantitative evidence, for example, eMBIs in cancer patients [13]. Together with this study, these results provide support for a larger, quantitative trial directly comparing eMBCT with group-based MBCT for cancer patients. Moreover, it would be interesting to directly compare individual eMBCT with individual group-based MBCT. Future trials should test for differences in treatment accessibility, program adherence, and treatment outcome between eMBCT with and without peer support, with and without synchronous communication modalities, and with and without therapist assistance [17]. This would allow us to further elucidate the predictors and mediators of treatment effect in Internet-based interventions [30] to help us determine which patient to offer group-based versus Internet-based treatment. Moreover, all of the abovementioned design alterations likely impact cost-effectiveness of the interventions, which should be considered [31]. Thus, future studies should preferably assess how different eMBCT delivery formats influence program adherence, mindfulness skills, and treatment outcome, and how different versions of eMBCT delivery formats compare both qualitatively and quantitatively with group-based MBCT.

### Strengths and Limitations

This is the first study to qualitatively explore facilitators and barriers of eMBCT for cancer patients. The relatively large sample size enabled us to reach data saturation and report a broad view of experiences. Moreover, we interviewed both completers and dropouts. Furthermore, we had the opportunity to gather data in the therapists. Nevertheless, our results should be interpreted within the limitations of our findings. We did not perform member checks to ensure validity of the verbatim transcripts. Moreover, the sample of the larger RCT consisted of cancer patients who self-selected themselves for a trial on an MBI. This implies that our findings cannot be extrapolated to cancer patients in general. In addition, some patients or therapists who participated in the training and were invited for focus groups or individual interviews declined participation, which may further limit the generalizability of our findings to all participating patients.

### Conclusions

We aimed to gain understanding of the facilitators and barriers of individual, asynchronous, and therapist-assisted eMBCT for cancer patients. Patients and therapists reported similar advantages and disadvantages of the timing, the individual nature, the asynchronous nature, the diaries, and the importance of self-discipline. Future studies should assess how different eMBCT delivery formats could further improve treatment accessibility, program adherence, and treatment outcome.

## Abbreviations

eMBCR: Internet-based mindfulness-based cancer recovery
eMBCT: Internet-based mindfulness-based cognitive therapy
eMBI: Internet-based mindfulness-based intervention
MBI: mindfulness-based intervention
MBCT: mindfulness-based cognitive therapy
MBSR: mindfulness-based stress reduction
RCT: randomized controlled trial
